# Global perspectives on monogenic forms of diabetes

**DOI:** 10.1007/s00125-025-06495-3

**Published:** 2025-07-16

**Authors:** James Russ-Silsby, Milena Teles, Samar S. Hassan, Nancy S. Elbarbary, Cấn Thị Bích Ngọc, Elisa De Franco

**Affiliations:** 1 Department of Clinical and Biomedical Science, University of Exeter Medical School, Exeter, UK; 2 Grupo de Diabetes Monogênico, Unidade de Endocrinologia Genética (LIM25), Unidade de Diabetes, Hospital das Clínicas, Faculdade de Medicina, Universidade de São Paulo, São Paulo, SP Brazil; 3 Núcleo de Pesquisa e Desenvolvimento de Medicamentos, Programa de Pós-Graduação em Medicina Translacional, Universidade Federal do Ceará, Fortaleza, Brazil; 4 Department of Pediatric Endocrinology, Gaafar Ibn Auf Pediatric Tertiary Hospital, Khartoum, Sudan; 5 Sudan Childhood Diabetes Center, Khartoum, Sudan; 6 Diabetes and Endocrine Unit, Department of Pediatrics, Faculty of Medicine, Ain Shams University, Cairo, Egypt; 7 Vietnam National Children’s Hospital, Hanoi, Vietnam

**Keywords:** Diagnosis, Equity, diversity and inclusion, Genetic testing, Global challenges, MODY, Monogenic diabetes, Neonatal diabetes, Penetrance, Review

## Abstract

Monogenic forms of diabetes represent an uncommon but very heterogeneous subset of the disease, with variable associated clinical features and key differences in treatment options. In this review, we discuss how advances in precision medicine and genomic sequencing have enhanced our understanding of the aetiology and clinical variability of monogenic diabetes. We highlight current global challenges, including the over-representation of individuals of European genetic ancestry in research studies, which complicates diagnosis in non-European populations, and national disparities in genetic testing strategies, which influence diagnostic accuracy. Additionally, we address issues in variant interpretation stemming from the increased understanding of variable penetrance in monogenic diabetes and the need to expand current reference datasets to exclude common genetic variation. Finally, we explore future directions, including the potential benefits of ongoing genetic studies for under-represented populations, the benefits and potential pitfalls of newborn screening programmes, and the potential of stem cell-derived islet transplantation and glucagon-like peptide- 1 receptor agonists as treatments for some forms of monogenic diabetes.

**Graphical Abstract:**

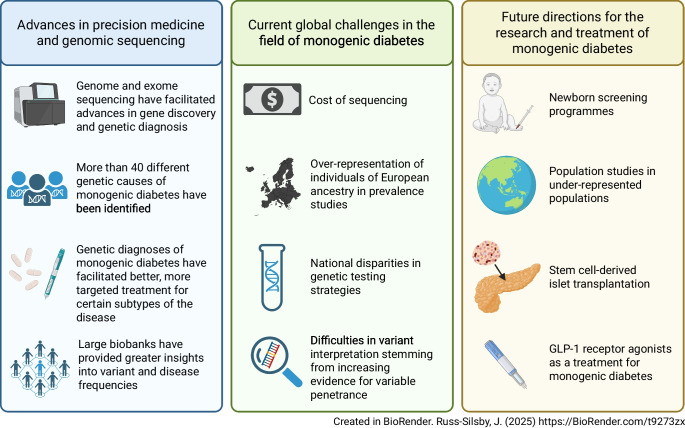

**Supplementary Information:**

The online version contains a slide of the figure for download available at 10.1007/s00125-025-06495-3.

## Introduction

**Fig. 1:**
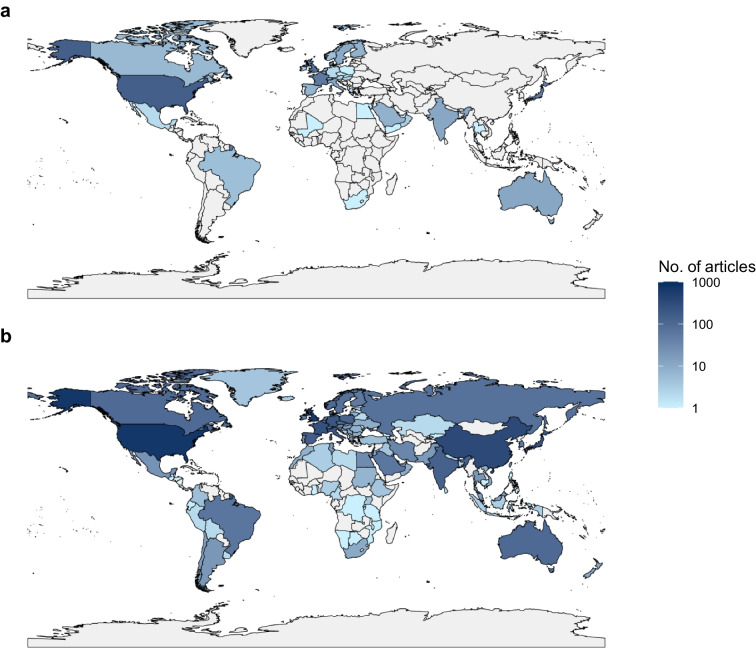
Number of scientific journal articles matching the terms ‘monogenic diabetes’, ‘maturity-onset diabetes of the young’ or ‘neonatal diabetes’ (and variations of these terms) in the Scopus database (https://www.scopus.com) up to 2000 (**a**) and from 2000 onwards (**b**) by country (based on all named author affiliations). Authors from 114 different countries have published research on monogenic diabetes since 2000 compared with authors from 41 countries prior to 2000, illustrating how, similar to other diseases, the study of monogenic diabetes has become an increasingly global field and why global perspectives on this disease are important. This figure is available as a downloadable slide

In the last 15 years, the study of monogenic diabetes subtypes, specifically neonatal diabetes and MODY, has increasingly become a global field (Fig. 1), strongly contributing to the understanding, diagnosis and management of individuals affected by these uncommon conditions. However, global challenges remain, including the identification of individuals who should be referred for genetic testing, testing strategies, gene discovery and genetic interpretation. In this review, we explore recent advances and future challenges in monogenic diabetes from a global perspective.

## Overview of the main monogenic diabetes subtypes

### Neonatal diabetes

#### Clinical features of neonatal diabetes

Neonatal diabetes mellitus (NDM) is defined as diabetes with onset in the first 6 months of life (see Text box, Summary of main monogenic diabetes subtypes). This clear diagnostic criterion is based on two studies showing that diabetes diagnosed before 6 months of age is most likely to have a monogenic cause rather than being due to polygenic autoimmunity [1, 2].


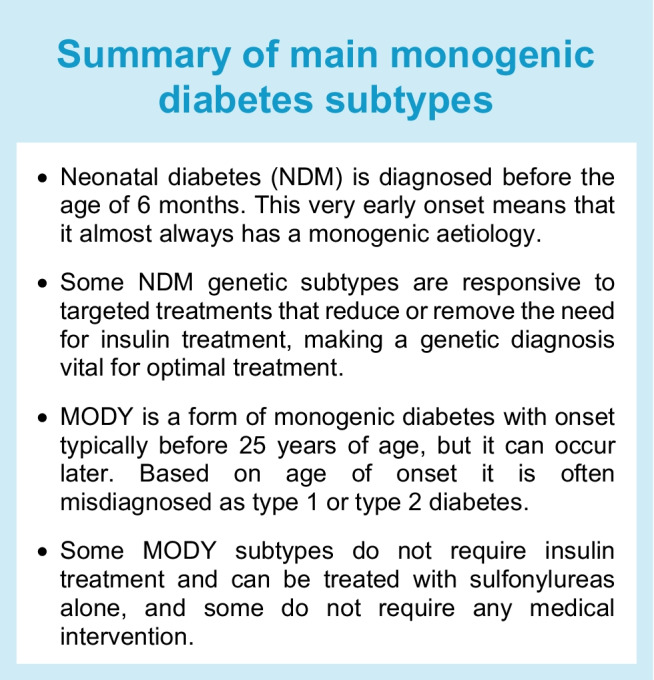


Clinically, NDM is categorised into three main types based on diabetes progression and additional extra-pancreatic features: permanent neonatal diabetes mellitus (PNDM), transient neonatal diabetes mellitus (TNDM) and syndromic neonatal diabetes [3]. Isolated PNDM is the most common type and requires lifelong therapy. TNDM is characterised by a period of remission in infancy, during which no treatment is required, but often relapses later in childhood or adolescence. Syndromic neonatal diabetes includes a range of conditions in which NDM is one of several clinical features, reflecting a broader systemic involvement [3]. Examples of syndromic NDM subtypes include pancreatic agenesis, clinically defined as NDM and exocrine pancreatic insufficiency [4], and IPEX (immune dysregulation, polyendocrinopathy, enteropathy, X-linked) syndrome, characterised by NDM, enteropathy and eczema [5].

#### Neonatal diabetes genetic testing

The International Society for Pediatric and Adolescent Diabetes (ISPAD) guidelines recommend genetic testing for all individuals diagnosed with diabetes in the first 6 months of life [6]. To date, 43 different genetic causes of NDM have been described, including autosomal dominant, autosomal recessive and X-linked variants, as well as an imprinting disorder and a form of aneuploidy [7].

#### Neonatal diabetes treatment

Identification of the genetic cause in individuals with NDM can result in improved treatment, highlighting the importance of precision medicine in the context of this disease (see Text box, Summary of main monogenic diabetes subtypes). Activating disease-causing variants in the genes encoding the ATP-dependent potassium (K_ATP_) channel, *KCNJ11* and *ABCC8*, are the most common cause of NDM [8]. Individuals with these variants often respond to treatment with sulfonylureas such as glibenclamide [9, 10] rather than insulin, leading to improved management of glucose levels [11]. When insulin treatment for NDM is required, advanced insulin delivery technologies such as insulin pumps and hybrid closed-loop systems have been shown to be safe and effective and can lead to improved management of glucose levels over standard insulin injections [12]. However, because of the cost of these therapies, they may not frequently be an option for individuals with NDM.

Beyond diabetes management, a genetic diagnosis of NDM can improve treatment for extra-pancreatic features. In the case of individuals with NDM caused by K_ATP_ channel gene variants, timely intervention with glibenclamide can significantly improve neurodevelopmental outcomes [11] with early treatment in affected individuals associated with reduced neurodevelopmental impairment and greater independence in later life [13].

### MODY

#### Clinical features of MODY

In contrast to NDM, there is no single defining criterion for diagnosis of MODY. Traditionally, MODY has been defined as a subtype of diabetes characterised by a strong family history and early age of onset (typically <25 years of age). However, heterozygous MODY-causing variants can occur de novo and so a family history of diabetes may not always be present. There is also increasing evidence from population databases and genetic testing of family members that onset of MODY after 25 years of age is not uncommon [14, 15]. Based on age at diagnosis, individuals with MODY are frequently misdiagnosed with either type 1 or type 2 diabetes [16] (see Text box, Summary of main monogenic diabetes subtypes).

#### MODY genetic testing

ISPAD guidelines recommend considering genetic testing for MODY in people with a family history of diabetes whose clinical features do not clearly align with classical type 1 or type 2 diabetes [6]. Additional indicators include low levels or absence of islet autoantibodies, preserved C-peptide levels years after diagnosis and features suggestive of specific MODY subtypes (e.g. mild stable fasting hyperglycaemia that does not progress in *GCK*-MODY, renal cysts in *HNF1B*-MODY or macrosomia in *HNF4A*-MODY) [17]. To aid the decision-making process around when MODY genetic testing is appropriate, MODY probability calculators based on family history of diabetes and clinical features are available [18, 19]. Strategies to identify individuals to refer for MODY testing have been reviewed elsewhere [17].

Monoallelic pathogenic variants in 11 genes are currently accepted as causative for MODY [20]. However, genetic testing panels for individuals with MODY will often include other genes, including those causing syndromic diseases that can include diabetes, such as Wolfram syndrome and mitochondrial maternally inherited diabetes and deafness (MIDD).

#### MODY treatment

Similarly to NDM, a genetic diagnosis of MODY is essential to ensure optimal clinical management, with the most common genetic subtypes not requiring insulin treatment [20] (see Text box, Summary of main monogenic diabetes subtypes). Individuals with pathogenic variants in the *HNF1A* and *HNF4A* genes usually respond well to sulfonylurea therapy, although requirement for insulin treatment later in life has been reported in some cases [21], while individuals with dominant *GCK* variants have mild fasting hyperglycaemia from birth that does not require any treatment and it is not associated with long-term complications [22, 23]. Some forms of MODY include additional extra-pancreatic features, such as those caused by monoallelic pathogenic variants in *HNF1B*, which cause renal cysts [24]. It is important that individuals with syndromic forms of diabetes receive a genetic diagnosis to ensure proper management of the extra-pancreatic features of the disease.

## Frequency of monogenic diabetes in different countries

Traditionally, NDM has been reported to affect 1 in 100,000 live births, while MODY is reported to affect 1 in 10,000 adults and 1 in 23,000 children (https://www.orpha.net/, accessed 5 Jun 2025). However, true disease prevalence can be difficult to accurately estimate, particularly in the case of NDM, where the genetic cause strongly influences life expectancy. Furthermore, the reported frequency of both diseases varies greatly in different populations. There are two primary explanations for this: population-specific differences in the frequencies of disease-causing variants and biases in clinical referrals for monogenic diabetes genetic testing [8, 25] (see Text box, Summary of frequency differences in monogenic diabetes).


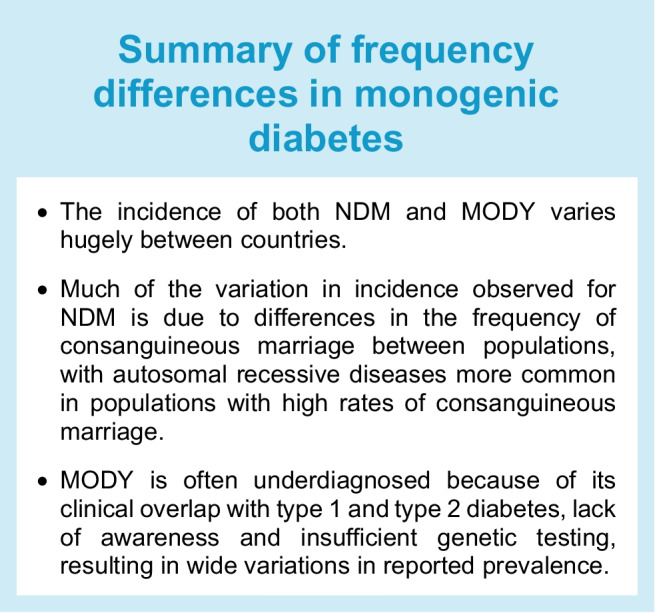


### Population-specific differences in disease incidence

The reported incidence of NDM ranges from as low as 1 in 476,000 (0.00021%) births in the USA to as high as 1 in 22,938 (0.0044%) in Qatar and 1 in 20,833 (0.0048%) in Sudan [26–28]. The biggest driver of this observed difference is the rate of consanguineous marriages; 27 of 43 known genetic causes of NDM are recessively acting [29] and are therefore more likely to occur in countries in which consanguineous marriage is common. Consistent with this, in countries with higher rates of consanguineous marriage the most common NDM subtypes are primarily recessive. For example, among some Arabic populations where the rate of consanguineous marriage is 40–60%, the most common genetic subtype of NDM is Wolcott–Rallison syndrome, caused by biallelic variants in the *EIF2AK3* gene [28, 30]. Conversely, in countries with low rates of consanguineous marriage the most common NDM subtypes have autosomal dominant inheritance, with heterozygous variants in *KCNJ11*, *ABCC8* and *INS* accounting for more than 50% of NDM cases in the USA and Europe [31, 32].

### Biases in referral rates

Biases in referral rates for monogenic diabetes testing are a key driver of the observed differences in the frequency of monogenic diabetes between countries. This is especially the case for MODY, where clinical overlap between MODY and type 1 and type 2 diabetes as well as a lack of awareness of the disease can lead to misdiagnosis and thus large variation in the reported frequency [25]. Within the UK it has been estimated that as many as 77% of MODY cases remain undiagnosed, with an estimated true case rate of 248 per million (0.025%) based on the prevalence in south-west England and Scotland, where disease awareness is high and similar prevalences have been reported [33]. A lower estimated prevalence has been reported in other European populations, such as the Netherlands (30 per million, 0.003% [34]) and Norway (92 per million, 0.0092% [35]), suggesting that similarly high proportions of cases may be undiagnosed.

To date, most studies on prevalence, genetics and clinical features of MODY have been conducted in European cohorts [25]. As a result, the prevalence of MODY in many non-European populations is currently unclear. This is further complicated by our current incomplete understanding of the differences in clinical features and genetic causes of MODY in non-European individuals, which impairs our ability to recognise the disease. Consequently, even within countries with higher referral rates such as the UK, significantly fewer non-European individuals are referred for MODY testing than would be expected based on the prevalence of diabetes in these groups [36].

## Genetic testing for monogenic diabetes

### Approaches to genetic testing

Traditionally, Sanger sequencing [37] of specific genes was used as the primary means of genetic testing for monogenic diabetes [8]. The development of next-generation sequencing and the discovery of more monogenic diabetes genes have led to replacement of this approach with methods that simultaneously test for all known genetic causes. However, Sanger sequencing is still the most affordable approach, especially for targeted testing in individuals with specific phenotypes (e.g. in cases of syndromic NDM) and as a first line of investigation to rapidly diagnose common genetic subtypes of monogenic diabetes that could result in treatment change.

Targeted next-generation sequencing (TNGS) of gene panels [38] enables sequencing of all genes and non-coding regions known to contain monogenic diabetes-causing genetic variants in a single assay [8] (see Text box, Summary of genetic testing in monogenic diabetes). The reduced cost of the assay compared with genome and exome sequencing, as well as the reduced data storage and processing costs, mean that TNGS has become one of the most popular methods for performing monogenic diabetes genetic testing worldwide. However, TNGS panels need to be constantly updated and redesigned as novel genetic aetiologies are identified.


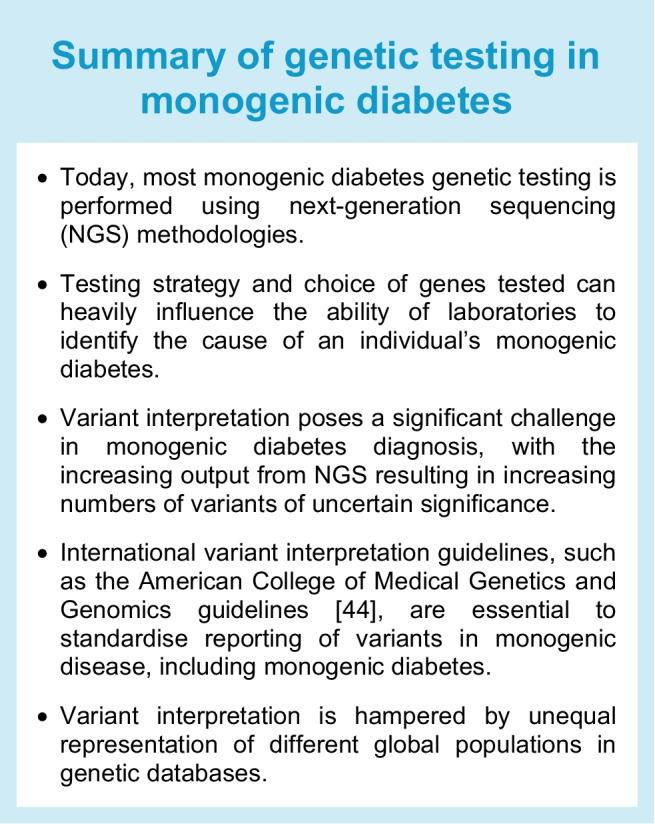


Analysis of known monogenic diabetes genes can be performed through exome (capturing all protein-coding genes) and genome (capturing nearly all the genome) sequencing [39]. Genome sequencing is the most comprehensive option for analysis of known genes and reanalysis of data when new aetiologies are identified, without the need for additional samples and laboratory work. However, the approximately 100-fold increase in number of variants observed in genome sequencing compared with exome sequencing makes the processing and prioritisation of variants more complex [40]. These complexities combined with the increased sequencing and data storage costs mean that genome sequencing is currently not frequently used for monogenic diabetes diagnostic testing.

### National differences in testing strategies

Different countries have adopted distinct testing strategies for monogenic diabetes based on healthcare infrastructure, funding and local expertise. While some countries have routine access to clinical exome or genome sequencing, others rely on more cost-effective approaches such as Sanger sequencing or TNGS. When local testing is not available, genetic analysis may sometimes be performed abroad at laboratories that accept international testing referrals, such as the Exeter Genomics Laboratory (https://www.diabetesgenes.org, accessed 5 Jun 2025), with charitable donations covering the costs of genetic testing of NDM when patients and their referring healthcare systems are unable to cover them. However, fewer funding opportunities exist for individuals with MODY from low-income countries.

The genetic testing strategy used heavily influences the rates of genetic diagnoses. When testing uses only Sanger sequencing or TNGS, the choice of genes tested is essential, as only variants in genes included in the test can be detected. Some genetic panels for MODY do not include genes causing syndromic forms of diabetes, such as *WFS1* (Wolfram syndrome 1) and *CISD2* (Wolfram syndrome 2) or the mitochondrial m.3243A>G variant causing MIDD, which may lead to missed diagnoses in cases where diabetes is the first manifestation of a syndrome [41]. Ideally, a consensus should be reached on the genes to be tested for all forms of monogenic diabetes. Similarly, the use of off-the-shelf exome sequencing assays that do not include non-coding regions can lead to missed diagnoses, as several non-coding regions have been implicated in monogenic diabetes [42]. This is a significant problem for individuals with NDM in countries such as Turkey, where non-coding variants in a distal regulatory element of the *PTF1A* gene are one of the leading causes of the disease [43].

### The challenges of variant interpretation in the genomics era

As next-generation sequencing techniques have enabled examination of increasingly large amounts of genetic variation in individuals with disease, variant interpretation has become more complex. While the implementation of guidelines from bodies such as the American College of Medical Genetics and Genomics [44] has been instrumental in moving the field towards a standardised method for variant assessment, huge challenges remain, with different laboratories often classifying variants differently, leaving clinicians and patients without clear guidance while waiting for more definitive answers. At worst, misinterpretation of a variant of uncertain significance can lead to unnecessary medical interventions or the failure to treat a disease properly. The use of resources such as ClinVar [45], which provides information on variant classification from different laboratories that have identified variants previously, is essential to improve consistency in variant classification.

A key piece of evidence used in variant interpretation is the frequency of a variant in affected and unaffected populations. As population databases of genetic variation are over-represented for individuals of European genetic ancestry [46], variant interpretation is more challenging in individuals of non-European genetic ancestry. This issue is actively being addressed, with several projects aimed at sequencing under-represented populations and global biobank initiatives currently under way (summarised in Table 1). Integration of genetic variation data from these projects into variant interpretation pipelines has the potential to vastly improve our ability to interpret rare variation in individuals of non-European genetic ancestry.

**Table 1:** Overview of large-scale sequencing projects currently under way in different populations

Project	Targeted group(s)	Projected sample size	Technology	Source
All of Us Research Program	Under-represented groups in the USA	>500,000	Array genotyping, genome sequencing	[79], https://allofus.nih.gov
OurDNA	Indigenous and non-European individuals in Australia	>7000	Genome sequencing	https://www.ourdna.org.au
Genes & Health	South Asian individuals in the UK	Up to 100,000	Array genotyping, exome sequencing	https://www.genesandhealth.org
Qatar Biobank	General Qatari population	60,000	Genome sequencing	[80]
Mexican Biobank	General Mexican population	6144	Array genotyping	[81]
Taiwan Biobank	General Taiwanese population	>150,000	Array genotyping	[82]

## Penetrance of monogenic diabetes and the monogenic–polygenic diabetes continuum

### Identification and implications of variable penetrance in monogenic diabetes subtypes

Increased understanding of the genetics underlying monogenic diabetes has highlighted the variability in penetrance of some genetic subtypes (see Text box, Summary of penetrance in monogenic diabetes). Understanding the mechanisms underlying this variability is important to guide genetic counselling.


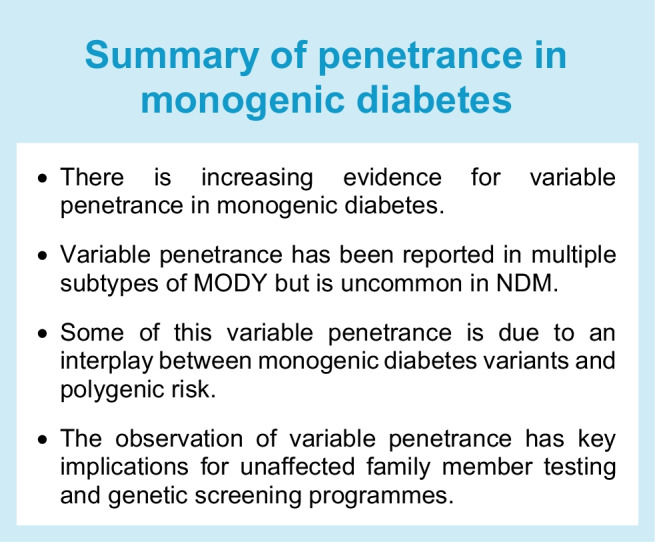


Variable penetrance has been especially well documented in MODY, with dominant *RFX6* loss-of-function variants and the hypomorphic *HNF4A* p.(Arg114Trp) variant reported to cause MODY with incomplete penetrance and variable expressivity [47, 48]. Recently, a study investigating the presence of disease-causing *HNF1A* and *HNF4A* variants in large non-clinically selected cohorts showed that penetrance of monogenic diabetes is overestimated based on data in clinically selected cohorts [49]. Variation in penetrance and expressivity of disease-causing variants adds additional complexity to the diagnosis of MODY, as it can lead to a weaker family history of diabetes and increased challenges in classifying possible disease variants, as they may still be present in population databases [37].

Variable penetrance appears to be less extreme in NDM, with disease-causing NDM variants being largely absent or ultra-rare in population databases. However, variability in age at diagnosis and disease features has been reported for some NDM subtypes. This could be variant dependent, as is the case for K_ATP_ variants, where the phenotypic spectrum depends on the severity of the variant effect on the channel [50, 51] or be due to stochastic variation, for example for *GATA6* variants, which have been reported to cause a wide spectrum of disease, from syndromic NDM to isolated adult-onset diabetes, even within the same family [52].

Variants within the mitochondrial genome add an additional layer of complexity to penetrance assessment and variant interpretation because multiple copies of the mitochondrial genome exist in every cell, ranging from hundreds to thousands of copies depending on the tissue [53]. This means that a mitochondrial variant can be present at variable levels, a phenomenon called heteroplasmy. The mitochondrial variant most commonly linked to monogenic diabetes is the m.3243A>G variant causing MIDD. The same variant, however, is also known to cause the more severe phenotype of mitochondrial encephalomyopathy with lactic acidosis and stroke-like episodes (MELAS) [54]. The level of heteroplasmy in the m.3243A>G variant has been shown to directly influence the phenotype [55]. A recent study observed that, in clinically unselected populations, mitochondrial variants, including m.3243A>G, are often present at low heteroplasmy levels. These variants were found to frequently have low penetrance, and penetrance and expressivity were highly correlated with the level of heteroplasmy [56].

### Polygenic contributions to monogenic diabetes penetrance and expressivity

Emerging evidence suggests that common polygenic risk alleles can modulate the expression and severity of monogenic disease [57]. This observation highlights the possibility of a continuum between strictly monogenic and polygenic diabetes, in which both rare, highly penetrant variants and more common, lower penetrance variants interact to shape the clinical phenotype. Preliminary evidence from studies using the UK Biobank and US BioMe biobank have shown significant associations between polygenic risk for type 2 diabetes and phenotype in individuals with rare monogenic diabetes variants, supporting a role for polygenic background in the penetrance and expressivity of MODY [58, 59].

An interplay between polygenic risk and monogenic diabetes has also been reported in monogenic autoimmune forms of diabetes, including subtypes presenting as NDM. Individuals with diabetes caused by pathogenic variants in the *AIRE*, *FOXP3*, *IL2RA*, *LRBA*, *STAT3* and *TNFAIP3* genes were found to have significantly higher type 1 diabetes genetic risk scores than healthy population-matched control individuals, while still having significantly lower scores than individuals with type 1 diabetes [60]. The mechanism by which the elevated risk influences development of autoimmune diabetes in individuals with these genetic subtypes is not yet understood.

### Implications of screening unaffected individuals

The variable penetrance of certain monogenic diabetes subtypes has key implications for the genetic counselling of individuals with disease-causing variants and their families. Furthermore, it draws into question the appropriateness of testing asymptomatic family members, as well as the general population (e.g. as part of the newborn screening programme being undertaken in the UK [61]), as individuals harbouring variable penetrance disease-causing variants may not develop the disease. A more complete understanding of the penetrance and features of different monogenic diabetes subtypes will be vital to reach a consensus on when to report disease-causing variants in unaffected individuals. This is essential to negate the risk of unnecessary treatments, while maximising the potential benefits of treating the disease before the onset of severe or potentially life-threatening complications.

## Future directions

### Improving equitability in access to genetic testing for monogenic diabetes

Despite improvements in the cost and availability of next-generation sequencing, global access to genetic testing remains highly inequitable. Two primary barriers are the availability of genetic testing facilities and a lack of awareness of monogenic diabetes among clinical staff in some areas. Education will play a key role in addressing these issues.

Online training courses in monogenic diabetes, such as those offered by the Royal Devon University Healthcare NHS Foundation Trust (https://www.diabetesgenes.org/training/), have already been highly successful in raising awareness [33] and attract attendees from around the world. However, their reach remains limited by language barriers and internet access. Expanding these courses to include in-person teaching in regions with lower awareness is vital to broadening their impact. Previously, the African Genomic Medicine Training Initiative [62] has successfully built a network of local trainers across Africa and trained thousands of doctors, nurses and scientists in genomic medicine. Replicating this model for monogenic diabetes training worldwide could greatly improve diagnostic equity. In addition to enhancing recognition of monogenic diabetes, such courses could also clarify options for genetic testing, including low-cost methods and available funding schemes.

Simplifying DNA sample collection could also significantly improve access to genetic testing. At present, DNA is primarily extracted from venous blood samples collected by primary healthcare workers; a process that can pose a major challenge for patients in remote regions, where the nearest clinic may be hours or even days away. This is additionally complicated by the need for DNA to be extracted within 7 days from venous blood sample collection. Optimising next-generation sequencing methods to use DNA from finger-prick capillary blood or saliva (akin to commercial direct-to-consumer genetic tests) would alleviate this barrier, as collection kits could be mailed directly to patients without requiring a healthcare worker’s attendance. Saliva samples have already been shown to produce sufficient DNA for genome sequencing in most cases [63] and preliminary data on capillary blood shows a similar success rate [64].

One of the greatest barriers to equitable monogenic diabetes testing is the limited understanding of its presentation in non-European populations [65]. This gap is especially problematic for conditions such as MODY, which are challenging to distinguish from type 1 and type 2 diabetes. Conducting more research in understudied populations is essential to characterise phenotypic differences and refine disease classification. This can be achieved through the establishment of large biobanks integrating genotypic and phenotypic information in non-European populations [66] and by publication of case reports of monogenic diabetes in these populations. Such insights should then be integrated into decision-support tools that are easily accessible by primary healthcare providers, such as the MODY calculator, which simplify the choice of when to pursue genetic testing but which currently underperform in individuals of non-European genetic ancestry [67].

### Undiagnosed individuals: gene discovery and polygenic phenocopies

Identifying the genetic causes of monogenic diabetes is essential to provide a genetic diagnosis and reveals essential information about the pathways that govern pancreatic beta cell development and function. By examining these pathways, we can gain insights that impact individuals with all forms of diabetes.

Despite comprehensive genetic testing, some individuals still lack a genetic diagnosis. At present, approximately 85% of individuals referred for NDM testing [68] and 25% of individuals referred for MODY genetic testing [36] receive a genetic diagnosis. Ongoing research seeks to discover additional disease genes and identify cases that represent polygenic or environmental phenocopies.

Polygenic type 1 and type 2 diabetes is likely to be the underlying diagnosis for some individuals referred for NDM and MODY testing. Extreme early-onset type 1 diabetes has been proposed as being responsible for approximately 4% of NDM cases [69]. The difficulty in distinguishing type 1 and type 2 diabetes from MODY means that many referred but genetically undiagnosed individuals may instead have polygenic diabetes. Calculation of the type 1 diabetes genetic risk score has been shown to be effective in discriminating monogenic and type 1 diabetes [70]. However, methods to exclude type 2 diabetes based on genetic/polygenic risk remain far from perfect, with known genetic variation explaining only approximately 20% of type 2 diabetes heritability [71].

The ability to sequence the complete exomes and genomes of individuals with monogenic diabetes using next-generation sequencing has facilitated a paradigm shift in gene discovery, moving from a candidate gene-based approach to one that is entirely gene agnostic [72]. This means that researchers no longer need large family trees or prior knowledge of specific genes to pinpoint pathogenic variants. This has led to the discovery of at least 16 new genetic causes of NDM and MODY, marking a significant expansion in our understanding of these diseases (Table 2). Further genetic causes are likely to remain unidentified, with the main challenge now being the identification of the contribution of variants in the non-coding genome.

**Table 2:** List of MODY and NDM aetiological genes identified through next-generation sequencing approaches

Gene/region^a^	Disease	Sequencing technology	Source
*KCNJ11*	MODY	Exome sequencing	[83]
*RFX6*	MODY	TNGS	[47]
*CNOT1*	NDM	Exome sequencing	[84]
*EIF2S3*	NDM	Exome sequencing	[85]
*FICD*	NDM	Genome sequencing	[86]
*GATA6*	NDM	TNGS	[4]
*LRBA*	NDM	TNGS	[87]
*NARS2*	NDM	Exome sequencing	[88]
*ONECUT1*	NDM	Genome sequencing	[89, 90]
*PD-L1* (*CD274*)	NDM	Genome sequencing	[91]
*PDIA6*	NDM	Genome sequencing	[92, 93]
*PTF1A* enhancer	NDM	Genome sequencing	[94]
*TARS2*	NDM	Genome sequencing	[95]
*WFS1*	NDM	Exome sequencing	[96, 97]
*YIPF5*	NDM	Genome sequencing	[98]
*ZNF808*	NDM	Genome sequencing	[99]

^a^Only genes with variants identified in at least three families are included

### Novel monogenic diabetes treatments

In recent years, several new treatments for different forms of monogenic diabetes have been proposed. In MODY, glucagon-like peptide-1 receptor agonists (GLP-1 RAs) such as dulaglutide and semaglutide have been highlighted as having strong therapeutic potential, with successful treatment reported in cases of *HNF1A*-, *HNF4A*- and *ABCC8*-MODY [73–75]. Early reports have also suggested that GLP-1 RAs may be effective for notoriously hard-to-treat syndromic forms of monogenic diabetes, such as *HNF1B*-MODY [76, 77]. These drugs, which have been shown to improve management of glucose levels and promote weight loss, are a promising new treatment for forms of monogenic diabetes in which some beta cell function is retained and are likely to continue to be trialled in different forms of monogenic diabetes.

When monogenic diabetes results from complete beta cell loss, transplantation of stem cell-derived islets is a promising new treatment option. The first successful application of this approach was recently reported in an individual with type 1 diabetes, with complete control of glucose levels from purely endogenous insulin detected at 75 days and no safety concerns after 1 year [78]. This exciting result highlights the potential of stem cell-derived islet transplantation to restore natural control of glucose levels and to revolutionise the treatment of subtypes of monogenic diabetes that currently can be treated only with exogenous insulin injections.

## Final remarks

Monogenic diabetes spans a broad spectrum of disorders presenting at various stages of life with diverse clinical features and treatment needs. While advances in genetic testing have improved our understanding of the genetic basis of the disease, challenges remain in distinguishing monogenic diabetes from the more common polygenic forms, particularly given incomplete penetrance, referral biases and the influence of polygenic risk alleles on monogenic disease presentation. The increased use of next-generation sequencing for genetic testing and the generation of large-scale genome data in non-clinical populations will be critical to further understand the genetic causes of monogenic diabetes, discover factors underlying variable penetrance and improve variant classification. These efforts are essential to ensure more accurate diagnoses, reduce misclassification and optimise care for individuals with these complex and uncommon forms of diabetes.

## Supplementary Information

Below is the link to the electronic supplementary material.

Supplementary file1 (PPTX 301 KB)

## Abbreviations

GLP-1 RA: Glucagon-like peptide-1 receptor agonist
MIDD: Maternally inherited diabetes and deafness
NDM: Neonatal diabetes mellitus
PNDM: Permanent neonatal diabetes mellitus
TNDM: Transient neonatal diabetes mellitus
TNGS: Targeted next-generation sequencing
