# Supplementary material for: Global perspectives on monogenic forms of diabetes
Source: Diabetologia. 2025 Jul 16;68(11):2362–73. doi: 10.1007/s00125-025-06495-3 (PMC12534271; doi:10.1007/s00125-025-06495-3)
Supplement: Supplementary file 1 — Supplementary file1 (PPTX 301 KB) [file 125_2025_6495_MOESM1_ESM.pptx]

## Slide 1
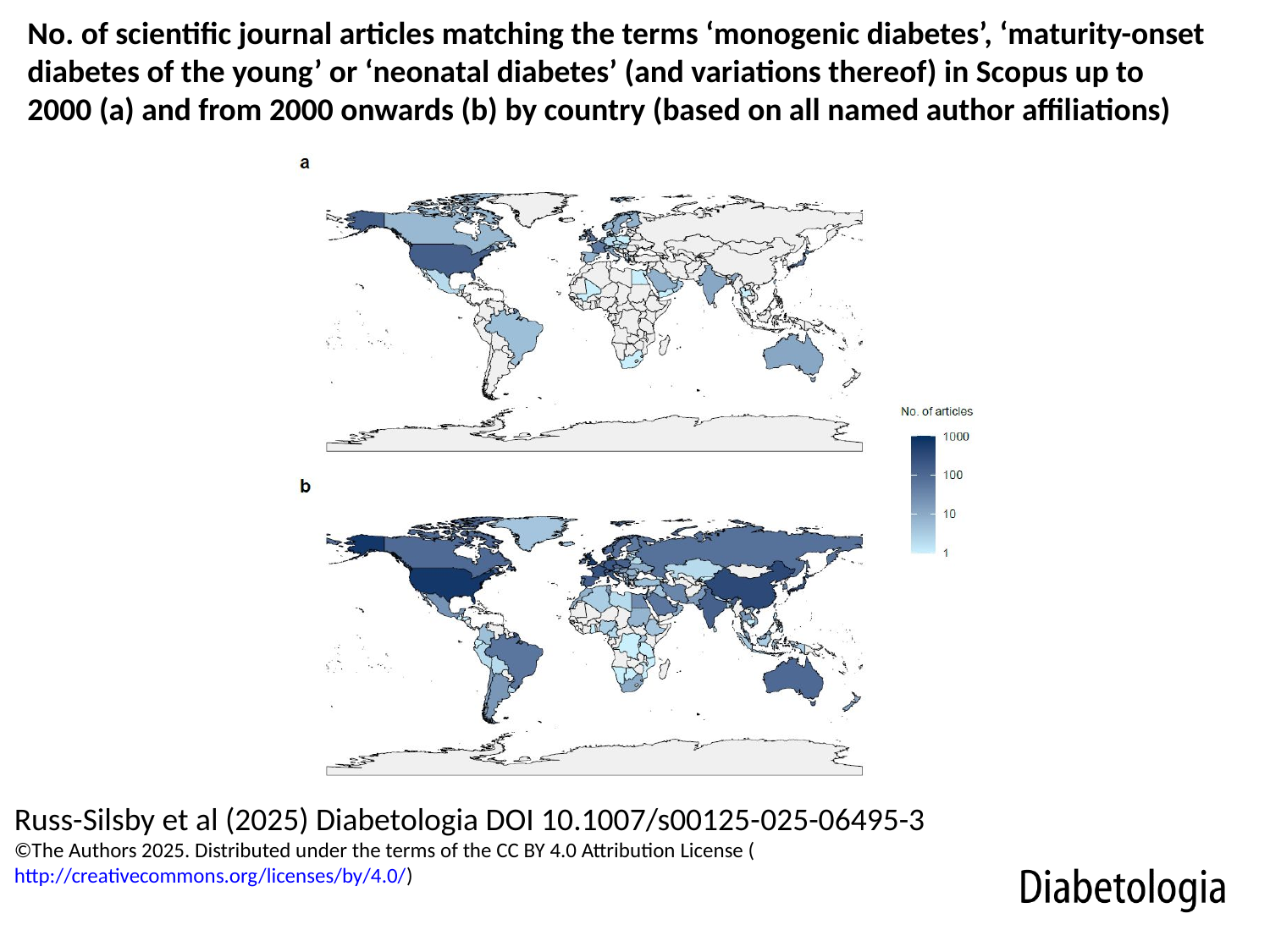

No. of scientific journal articles matching the terms ‘monogenic diabetes’, ‘maturity-onset diabetes of the young’ or ‘neonatal diabetes’ (and variations thereof) in Scopus up to 2000 (a) and from 2000 onwards (b) by country (based on all named author affiliations)
Russ-Silsby et al (2025) Diabetologia DOI 10.1007/s00125-025-06495-3
©The Authors 2025. Distributed under the terms of the CC BY 4.0 Attribution License (http://creativecommons.org/licenses/by/4.0/)
